# Case series: oncologic patients in remission with giant ventral hernia treated with botulinum toxin and component separation

**DOI:** 10.1093/jscr/rjag013

**Published:** 2026-01-27

**Authors:** Luis Muñoz-Andrade, Francisco Nevárez, Sandra Chalén, Yanalín Mantuano

**Affiliations:** Department of Oncology Surgery, National Oncology Institute SOLCA Guayaquil, Guayaquil, 090505, Ecuador; Department of Digestive Surgery, National Oncology Institute SOLCA Guayaquil, Av. Pedro Menéndez Gilbert, Guayaquil, Guayas 090505, Ecuador; Department of Digestive Surgery, National Oncology Institute SOLCA Guayaquil, Av. Pedro Menéndez Gilbert, Guayaquil, Guayas 090505, Ecuador; Department of Plastic Surgery, National Oncology Institute SOLCA Guayaquil, Av. Pedro Menéndez Gilbert, Guayaquil, Guayas 090505, Ecuador; Department of Plastic Surgery, National Oncology Institute SOLCA Guayaquil, Av. Pedro Menéndez Gilbert, Guayaquil, Guayas 090505, Ecuador

**Keywords:** ventral hernia, botulinum toxin, component separation, abdominal wall reconstruction, oncology, loss of domain

## Abstract

Giant ventral hernia represents a surgical challenge, particularly in oncologic patients in remission, due to large fascial defects and loss of domain. Botulinum toxin has been incorporated as an adjuvant to facilitate fascial closure through chemical relaxation of the abdominal wall. We describe four oncologic patients in remission with giant ventral hernia treated with preoperative botulinum toxin and repair using component separation with retromuscular polypropylene mesh placement. The procedures were performed without intraoperative complications or early recurrence. The technique allowed tension-free fascial closure with favorable postoperative outcomes. Botulinum toxin appears to be a useful and safe tool for the repair of complex ventral hernias in oncologic patients in remission, promoting fascial approximation and reducing the need for more invasive procedures.

## Introduction

Complex ventral hernia remains one of the most demanding challenges in abdominal wall surgery. The magnitude of the defect, frequent loss of domain, and high recurrence risk make its management technically difficult and often associated with morbidity. In oncologic patients in remission, these difficulties are amplified by multiple laparotomies, visceral resections, and postoperative adhesions, increasing operative complexity and perioperative risk [1, 2].

During the last decade, the introduction of botulinum toxin type A (BTA) as a method of *chemical component separation* has emerged as an innovative adjunct in abdominal wall reconstruction. This pharmacologic approach induces temporary flaccid paralysis of the external, internal oblique, and transversus abdominis muscles, allowing the abdominal wall to elongate and the fascial edges to approximate without excessive tension [3, 4]. Such preoperative ‘chemical release’ facilitates closure in large and giant hernias, particularly those with severe loss of domain or scarring [5, 6].

A growing body of evidence, including prospective studies and systematic reviews, supports BTA as a safe and effective adjunct. Reported benefits include reduced need for traditional component separation, lower intra-abdominal pressure, fewer respiratory complications, and recurrence rates comparable, or inferior to standard procedures [7–9]. Combined with retromuscular polypropylene mesh reinforcement, it offers durable results and improved abdominal wall compliance, as validated by reference centers [10–12].

Recently, BTA has been applied in increasingly complex reconstructions—combined with progressive preoperative pneumoperitoneum to enhance abdominal domain, and in patients with prior abdominal or pelvic malignancy where fibrosis limits wall flexibility [13]. These studies show that BTA not only facilitates fascial closure but also improves respiratory function and recovery.

Despite growing evidence, data focusing on oncologic patients in remission remain limited. This subgroup often presents complex anatomical alterations and fragile tissue after extensive procedures. Documenting such cases is crucial to expand current knowledge and optimize abdominal wall reconstruction strategies in cancer survivors [14, 15].

## Case series

Four oncologic patients in remission were included—three men and one woman—aged between 43 and 66 years, all of whom developed giant ventral hernias following major abdominal surgeries performed for malignant disease. Each patient was evaluated in the outpatient surgical oncology clinic and selected for abdominal wall reconstruction using component separation and retromuscular polypropylene mesh placement, preceded by BTA injection as part of preoperative abdominal wall preparation.

### Case 1

**Figure 1:**
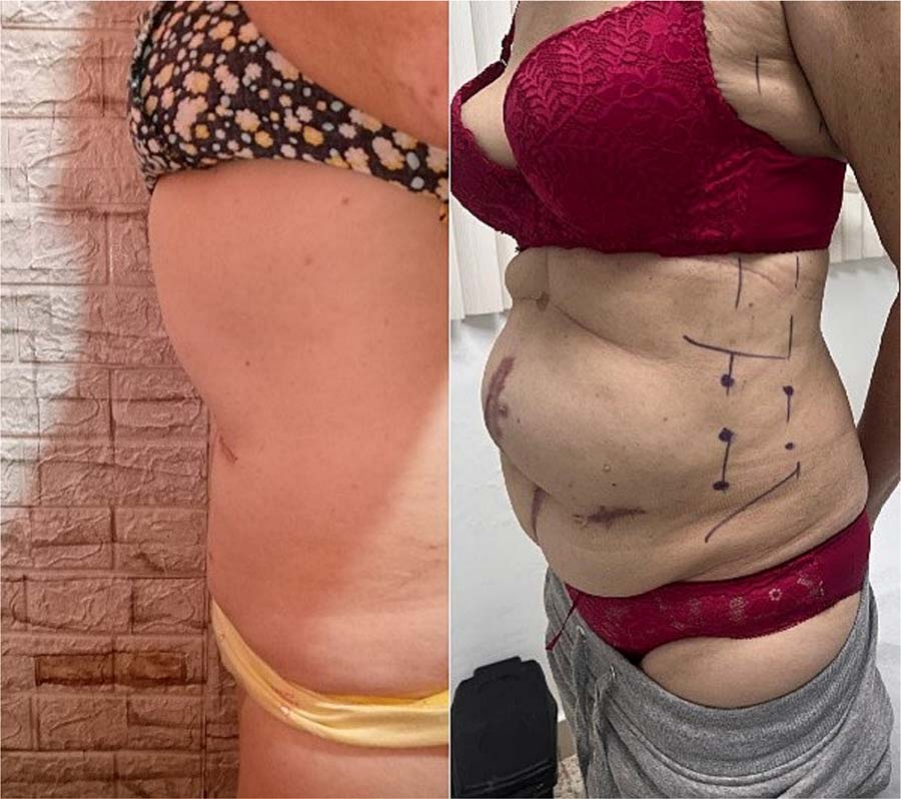
On the right, patient with preoperative marking for botulinum toxin application; on the left, postoperative appearance after component separation assisted by botulinum toxin.

A 55-year-old female with a history of sigmoid colon cancer treated by left hemicolectomy and subsequent colostomy closure developed a large ventral defect measuring approximately 23 cm, associated with loss of domain. She received a total of 100 IU of BTA, distributed bilaterally at ten injection sites, 1 month prior to surgery. A component separation was performed with retromuscular polypropylene mesh placement (15 × 30 cm). The operative time was 220 minutes. Postoperative recovery was uneventful, with no wound complications or recurrence at 12-month follow-up (Fig. 1).

### Case 2

**Figure 2:**
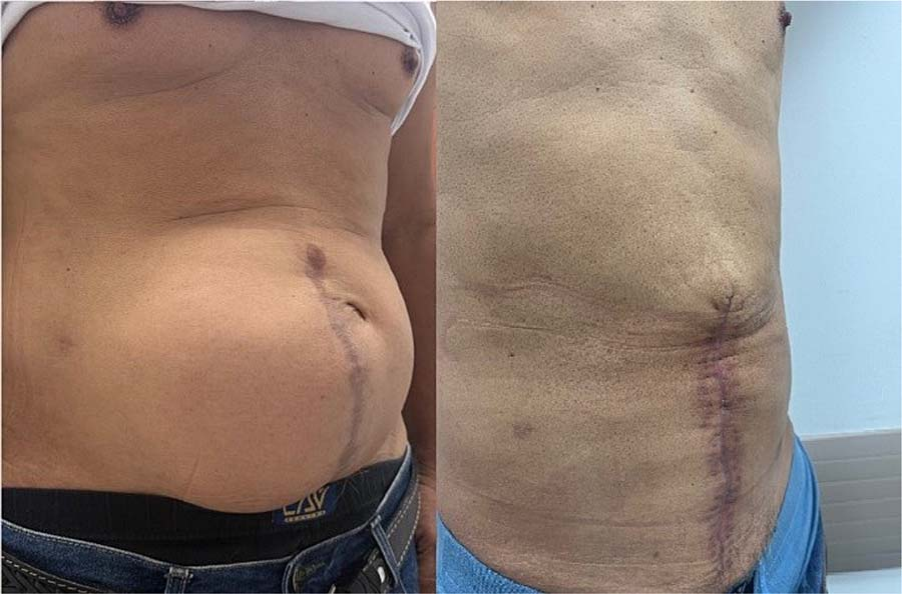
Preoperative image (left) of a patient with a 19 cm ventral hernia; on the right, postoperative result after repair with component separation and botulinum toxin use.

A 66-year-old male with a history of poorly differentiated adenocarcinoma of the colon, previously treated with a right hemicolectomy in October 2024, developed a 22 cm ventral hernia. A total of 90 IU of BTA was injected at six sites (15 IU each) three weeks before surgery. The patient underwent component separation with polypropylene mesh repair. The operative time was 140 minutes. He experienced an uncomplicated postoperative course and remains recurrence-free on follow-up (Fig. 2).

### Case 3

**Figure 3:**
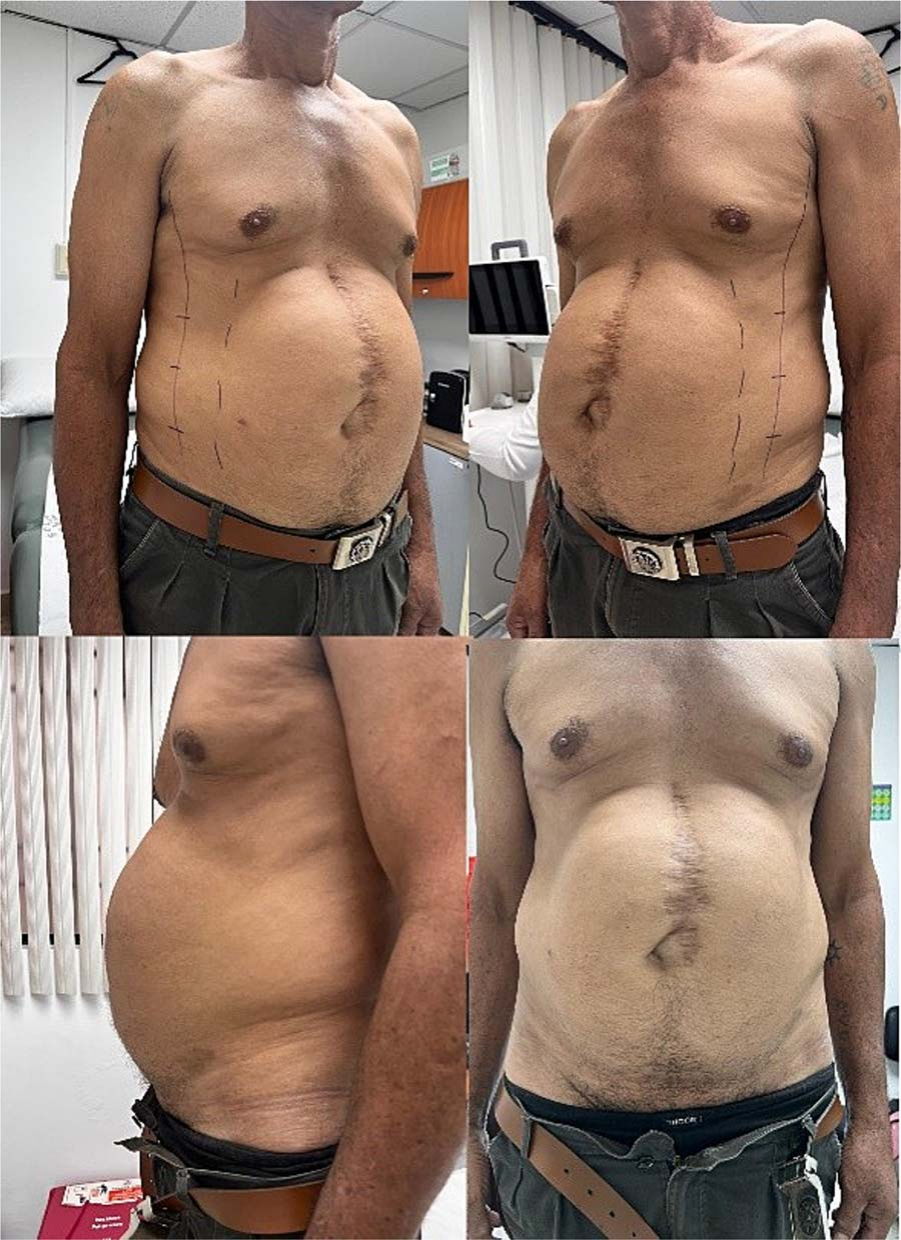
Patient with a giant ventral hernia larger than 23 cm, prepared for botulinum toxin application as a preoperative strategy to facilitate abdominal wall closure.

A 65-year-old male with a history of mucinous adenocarcinoma of the ascending colon, treated by right hemicolectomy, presented with a ventral defect measuring between 18 and 21 cm. He received BTA injection 1 month before the procedure (Fig. 3). The operation involved component separation and polypropylene mesh reconstruction in an ‘H’ configuration. The operative time was 140 minutes. No perioperative complications occurred, and the patient continues recurrence-free at 6-month follow-up.

### Case 4

**Figure 4:**
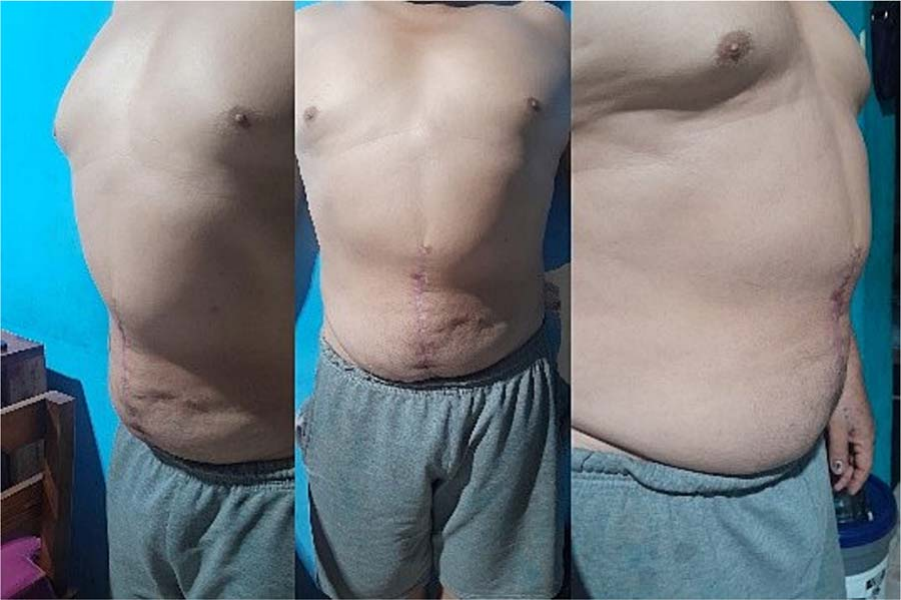
Postoperative result of a patient who underwent component separation with adjuvant botulinum toxin application, showing improved abdominal wall integrity.

A 43-year-old male with a history of bladder tumor and multiple prior laparotomies—including partial cystectomy, sigmoidectomy, rectovesical fistula repair, colostomy, and later closure—developed a giant ventral hernia measuring approximately 19 cm. He received BTA injection 1 month prior to surgery. Reconstruction was performed using component separation with retromuscular polypropylene mesh placement. The postoperative course was favorable and free of complications, and the patient remains in good general condition under ambulatory follow-up (Fig. 4).

In all four patients, botulinum toxin administration effectively increased abdominal wall compliance, allowing tension-free fascial closure and facilitating primary repair without the need for more extensive or invasive release techniques. None of the cases showed intraoperative or major postoperative complications, and no early recurrences have been observed to date.

### Preoperative botulinum toxin administration

All patients received BTA injections under ultrasound guidance approximately four weeks prior to surgery, as part of a standardized preoperative protocol designed to achieve controlled chemical relaxation of the lateral abdominal wall muscles.

**Figure 5:**
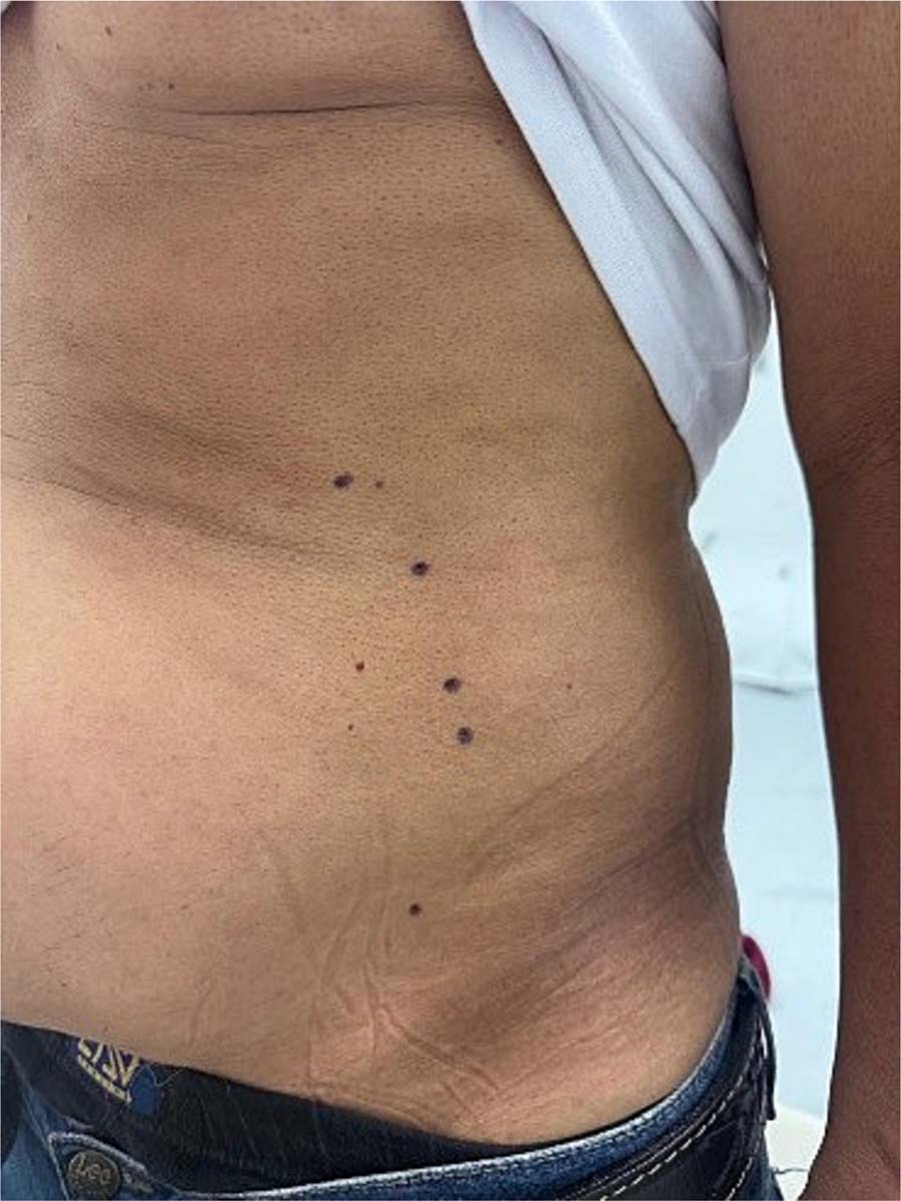
Marking on the abdominal wall of a patient candidate for botulinum toxin injection prior to ventral hernia repair.

1. Each patient was positioned in the supine position on the operating table.
2. Local asepsis was performed, and injection sites were marked on the abdominal wall (Fig. 5).
3. Under real-time ultrasound guidance, the external oblique, internal oblique, and transversus abdominis muscles were clearly identified to ensure accurate needle placement.
4. Fractionated doses totaling 90–100 IU of BTA were injected bilaterally at multiple points, ensuring uniform intramuscular infiltration along the muscle bellies.
5. No local or systemic complications related to the injection procedure were observed.

The main objective of this intervention was to achieve chemical denervation and relaxation of the lateral abdominal muscles, thereby increasing their elasticity and compliance. This preoperative elongation of the abdominal wall facilitates tension-free fascial closure during the definitive hernia repair procedure.

### Component separation and mesh placement

Approximately 1 month after botulinum toxin administration, all patients underwent elective abdominal wall reconstruction under general anesthesia. The following standardized steps were performed:

1. A midline incision was made along the previous surgical scar, followed by meticulous lysis of adhesions to expose the fascial edges.
2. The borders of the fascial defect were identified, with measured diameters ranging from 10 to 14 cm.
3. Component separation was then performed by releasing the lateral abdominal wall muscles, following the standard Ramírez technique, to achieve adequate medial mobilization.
4. A satisfactory medial advancement of the muscular flaps was obtained, allowing primary fascial closure without tension.
5. A retromuscular (sublay) polypropylene mesh of appropriate size was placed to reinforce the repair and maintain abdominal wall integrity.
6. The mesh was secured with continuous non-absorbable sutures, and the midline fascia was closed in a single continuous layer.
7. Closed-suction drains were placed when deemed necessary to prevent seroma or hematoma formation.

## Discussion

Repair of giant ventral hernia in oncologic patients in remission represents a major and technically demanding surgical challenge. These patients often have a history of multiple laparotomies, postoperative scarring, and distortion of the abdominal wall anatomy. As a result, they tend to develop large fascial defects associated with loss of domain, making tension-free fascial closure difficult and increasing the risk of complications such as wound dehiscence, respiratory compromise, or recurrence [1, 2].

Within this scenario, the introduction of BTA as a preoperative strategy has become a significant advance in abdominal wall surgery. By inducing a temporary paralysis of the lateral abdominal muscles, BTA produces controlled elongation and tension reduction, facilitating primary fascial closure while minimizing the need for extensive surgical release [3, 4].

Recent series and meta-analyses have shown that preoperative BTA can improve respiratory outcomes and reduce postoperative morbidity in patients with large or complex hernias [5, 6]. In the literature, multiple authors have supported BTA as an adjunct to component separation. Timmer et al. reported in a meta-analysis that BTA significantly increases lateral abdominal wall length, reducing fascial tension [1].

Deerenberg *et al.* confirmed that it enhances closure feasibility and lowers recurrence rates [2]. Our experience aligns with these findings, as all four patients in our series achieved complete fascial closure without early recurrence. Another important consideration is the safety profile of BTA. Although occasional cardiopulmonary complications have been reported in patients with massive hernias and ventilatory compromise [5], no adverse events occurred in our cohort. This likely reflects that our patients were in oncologic remission and in good general condition at the time of surgery.

The preoperative use of BTA is indicated in patients with large or complex ventral hernias, particularly with defects >10 cm or loss of domain. It is also appropriate in cases with multiple laparotomies, extensive adhesions, or lateral wall fibrosis, where muscle compliance is reduced. In selected patients, BTA may reduce or replace the need for surgical component separation [8]. Additional indications include those with cardiopulmonary risk, since BTA improves abdominal compliance and facilitates gradual cavity adaptation. When combined with retromuscular mesh reinforcement, it contributes to safer fascial approximation.

Contraindications include neuromuscular disorders, pregnancy, and hypersensitivity to the toxin. The combined approach of BTA injection followed by component separation and retromuscular mesh placement has produced favorable results in multiple publications [7–9]. Bueno-Lledó *et al.* showed that BTA alone can sometimes eliminate the need for component separation, allowing tension-free closure with shorter operative times [8].

In our experience, all patients required component separation, but BTA clearly facilitated fascial approximation and reduced technical strain. In more demanding cases, such as giant hernias with severe loss of domain, authors have proposed combining BTA with progressive pneumoperitoneum to further expand the abdominal cavity and improve elasticity [13]. Although this technique was not used in our series, evidence supports its value in extreme scenarios.

The subgroup of oncologic patients in remission deserves special attention. These individuals often have multiple scars, dense adhesions, and altered tissue quality, increasing surgical risk. Nevertheless, BTA has proven effective and safe in this population. Mandujano *et al.* and Serafío-Gómez *et al.* documented successful closures with low complication rates [14, 15]. Our results reinforce these observations, demonstrating reproducibility and safety in patients with digestive or urological malignancies. Overall, current evidence supports BTA as an effective, low-risk, and reproducible adjunct for giant or complex ventral hernia repair.

Our findings are consistent with previous studies; the main limitations are the small sample size and short follow-up. Nevertheless, this series adds valuable information on oncologic patients in remission, a group still underrepresented in the literature, and highlights the potential of BTA to optimize fascial closure and surgical outcomes.
